# Mechanism of Chronic Kidney Disease Progression and Novel Biomarkers: A Metabolomic Analysis of Experimental Glomerulonephritis

**DOI:** 10.3390/metabo10040169

**Published:** 2020-04-24

**Authors:** Kyoung Hee Han, Bora Kim, Sang Chun Ji, Hee Gyung Kang, Hae Il Cheong, Joo-Youn Cho, Il-Soo Ha

**Affiliations:** 1 Department of Pediatrics, Jeju National University School of Medicine, Aran 13gil 15, Jeju-si, Jeju 63241, Korea; hansyang78@gmail.com; 2 Kidney Research Institute, Seoul National University College of Medicine, Seoul 03080, Korea; borakim1234@gmail.com (B.K.); scji@snu.ac.kr (S.C.J.); kanghg1@gmail.com (H.G.K.); 3 Clinical Pharmacology and Therapeutics, Seoul National University College of Medicine and Hospital, 101 Daehak-ro, Jongno-gu, Seoul 03080, Korea; 4 Department of Pediatrics, Seoul National University College of Medicine and Hospital, 103, Daehak-ro, Jongno-gu, Seoul 03080, Korea; cheonghi@snu.ac.kr; 5 Department of Biomedical Sciences, Seoul National University College of Medicine, 101 Daehak-ro, Jongno-gu, Seoul 03080, Korea

**Keywords:** untargeted metabolomics, chronic glomerulonephritis, chronic kidney disease, experimental

## Abstract

While a complex network of cellular and molecular events is known to be involved in the pathophysiological mechanism of chronic kidney disease (CKD), the divergence point between reversal and progression and the event that triggers CKD progression are still unknown. To understand the different mechanisms between reversible and irreversible kidney disease and to search for urinary biomarkers that can predict prognosis, a metabolomic analysis was applied to compare acute and chronic experimental glomerulonephritis (GN) models. Four metabolites, namely, epoxyoctadecenoic acid (EpOME), epoxyeicosatetraenoic acid (EpETE), α-linolenic acid (ALA), and hydroxyretinoic acid, were identified as predictive markers after comparing the chronic nephritis model with acute nephritis and control groups (false discovery rate adjusted *p*-value (q-value) < 0.05). Renal mRNA expression of cytochrome P450 and epoxide hydrolase was also identified as being involved in the production of epoxide metabolites from these polyunsaturated fatty acids (*p* < 0.05). These results suggested that the progression of chronic kidney disease is associated with abnormally activated epoxide hydrolase, leading to an increase in EpOME and EpETE as pro-inflammatory eicosanoids.

## 1. Introduction

Chronic kidney disease (CKD) has a high worldwide prevalence, with drastically increased cases of end-stage renal disease [1]. CKD patients are clinically asymptomatic; hence, its clinical management is based on biomarkers of renal function and damage [2]. However, biomarkers that help predict a high risk of progression towards renal failure are lacking. Certain studies have reported advancements in the early detection and therapeutic intervention to decelerate CKD progression [3]. Edwards and Whyte (1959) reported a method involving the measurement of serum creatinine levels, thus providing a better indicator of renal function than methods based on the measurement of serum urea levels [4]. Cockcroft and Gault (1976) reported a method of estimating the glomerular filtration rate from serum creatinine levels [5]. Apart from creatinine, cystatin C is a well-known biomarker of renal function and CKD progression [6]. Creatinine and cystatin C are endogenous substrates and are commonly estimated in plasma and considered in the determination of the estimated glomerular filtration rate [7]. Moreover, albuminuria formation precedes the decline in renal function, hence serving as a marker of renal damage and is strongly associated with CKD progression [2]. Recent studies on biomarkers for the early detection of acute kidney injury (AKI) [8] have reported several biomarkers in experimental ischemic renal injury and clinical AKI, such as cystatin C [9], interleukin-18 [10], and neutrophil gelatinase-associated lipocalin [11], which result in a 50% increase in serum creatinine levels [12].

The kidney metabolizes numerous substrates. Glomerular filtration, tubular reabsorption, and secretion are involved in urine formation. Therefore, urine can provide important information regarding kidney function during certain pathologic conditions associated with the urinary system and normal physiologic changes [13,14]. Gao proposed a road map of urinary biomarkers in the early stages of kidney disease through a series of studies [15,16].

Recently, metabolomics has emerged as an important technology to measure small-molecule metabolites in tissues and biofluids [17]. The metabolome refers to the evolution of the genome, transcriptome, and proteome, reflecting real-time processes in living organisms [16]. Metabolomics is the study of chemical processes involved in the production of metabolites as downstream products derived from the genome [15]. Serum oxylipin profiles and metabolites associated with polyunsaturated fatty acids (PUFAs) based on metabolomics reflect alterations in renal function in patients with IgA nephropathy [18]. Moreover, 5-methoxytryptophan, levels of which are markedly associated with clinical CKD markers, has been identified through untargeted metabolomics among patients with Stage 1–5 CKD [19]. However, in most cases, metabolomics has been widely used to investigate the pathophysiology of renal fibrosis using only different animal models of CKD, such as 5/6 nephrectomized rats, adenine-induced CKD, and other drug-induced CKD [20,21,22,23,24]. However, these models do not reflect clinical conditions underlying the gradual progression of glomerular disease to CKD. 

Commonly used animal models of anti-Thy1 nephritis present with reversible mesangial proliferative glomerulonephritis, facilitating the assessment of the acute phase of glomerular disease. In this model, anti-Thy1 antibody immediately binds to mesangial cell receptor, causing mesangiolysis associated with transient complement activation and immune complex formation, which are immediately obliterated through mesangial proliferation to repair the glomerular damage due to mesangiolysis [25]. However, heminephrectomy can induce irreversible progressive glomerulosclerosis with crescent formation in anti-Thy1 nephritis [26].

Marked proteinuria of >1.0 g/d and severe histologic changes are associated with renal outcomes in IgA nephropathy in humans [27,28]. Moreover, the cumulative excretion of urinary podocytes differs between acute and chronic IgA nephropathy and Henoch–Schönlein purpura nephritis, thus reflecting disease progression [29]. Therefore, we conducted a non-targeted metabolomics animal study using urine specimens, assuming that the excreted urinary metabolites reflect differences in the underlying mechanism. Therefore, this study aimed to investigate the differences in the pathogenesis of acute and chronic nephritis and identify urinary metabolites predicting the progression or regression of kidney injuries through an untargeted metabolomics approach, using an experimental anti-Thy1.1-initiated model of mesangioproliferative glomerulonephritis with or without heminephrectomy in rats. We hypothesized that there is a quantitative difference of proteinuria between acute and chronic nephritis, and that qualitative differences in proteinuria cause pathological differences between acute and chronic nephritis. In other words, the metabolites of urine, which cause these pathological consequences, reflect the difference between acute and chronic nephritis, and thus these substances can be considered as a risk factor for CKD progression.

## 2. Material and Methods

### 2.1. Definition

**Figure 1:**
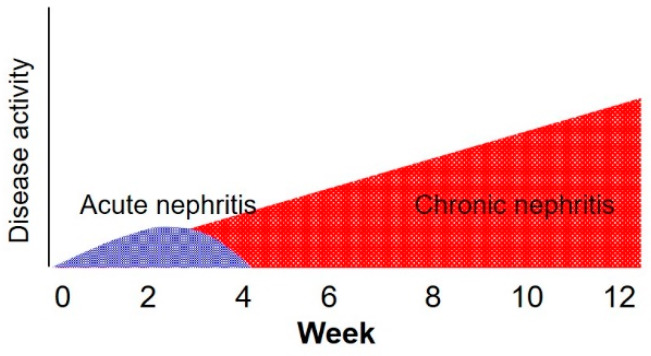
Schematic illustration of the definition of acute and chronic nephritis.

Acute nephritis (AN) is a sudden inflammation of the kidneys that affects renal function. When treated early, AN is usually temporary and reversible, leading to clinical improvement in about one month. AN can have a progressive tendency to chronic nephritis (CN). CN is characterized by irreversible pathologic findings, including interstitial fibrosis (IF), and if it lasts longer than three months, it leads to permanent loss of renal function; that is, CKD (Figure 1) [30,31].

### 2.2. Animals

Female Sprague Dawley rats (weight, 160–260 g) were provided by OrientBio, Seongnam-si, Korea. All experiments were approved by the Institutional Animal Care and Use Committee of the Seoul National University Hospital and animals were maintained in a facility accredited by the Association for Assessment and Accreditation of Laboratory Animal Care International (AAALAC International) (#001169) in accordance with the guide for the care and use of laboratory animals [32]. The animals were housed at the animal center (temperature: 20–26 °C; humidity: 50% ± 20%) and fed with Pico5053 (Pico Lab-Rodent 20-IRR, OrientBio, Seongnam-si, Korea) with free access to drinking water. 

### 2.3. Experimental Design

Acute anti-Thy 1.1 nephritis as a rat model of mesangial proliferative glomerulonephritis has been described previously [33]. Briefly, 8-week-old female rats were injected with an intravenous injection of anti-Thy1.1 antibody (Young in Frontier, Seoul, Korea). The development of progressive nephritis following a single injection anti-Thy1.1 antibody Ox-7 after unilateral nephrectomy in rats has been reported previously [26,34,35].

**Figure 2:**
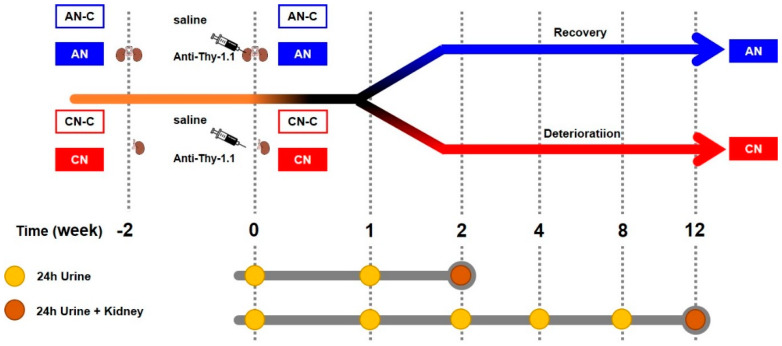
Experimental design of the nephritis rat model. The AN animals were produced via the injection of mouse anti-Thy1.1 antibody. The CN group was induced through the administration of the mouse anti-Thy1.1 antibody to unilaterally nephrectomized rats. AN, acute nephritis; AN-C, control group for acute nephritis; CN, chronic nephritis; CN-C, control group for chronic nephritis.

The experimental design is outlined in Figure 2. Forty specific pathogen-free Sprague Dawley rats were randomly divided into four groups as follows: AN (*n* = 12), CN (*n* = 12), control group for AN (AN-C, *n* = 8), and control group for CN (CN-C, *n* = 8). The AN group received a sham operation 2 weeks before the intravenous injection of 5 mg/kg of a mouse anti-Thy1.1 antibody via the tail vein on day 0. The CN group received heminephrectomy 2 weeks before intravenous injection of 5 mg/kg of the mouse anti-Thy1.1 antibody on day 0. The AN-C group received a sham operation 2 weeks before the intravenous injection of 5 mL/kg of PBS, whereas the CN-C group received heminephrectomy 2 weeks before the injection of 5 mL/kg of PBS on day 0.

Half of the rats were sacrificed at the end of 2 weeks (2W) and the other half were sacrificed at the end of 12 weeks (12W). Twenty-four-hour urine was obtained on Day 0, and at the end of Week 1, 2, 4, 8, and 12; thus, until sacrifice. All animals were anesthetized with a single intraperitoneal injection of 5 mg/kg xylazine and an intramuscular injection of 10 mg/kg zoletil before sacrifice [36,37,38].

### 2.4. Measurement of Proteinuria

Urinary protein concentrations were measured by the pyrogallol red–molybdate method (Randox Laboratories Ltd., Crumlin, UK). Creatinine levels were determined by an IDMS reference measurement procedure (Jaffe method) [39]. Proteinuria was expressed as the urine protein-to-creatinine ratio (mg/mg).

### 2.5. Evaluation of Renal Histology

Kidney sections were processed and examined by light microscopy (Leica DF280, Leica Microsystems, Wetzlar, Germany), as described previously [40]. Kidneys were perfused with cold PBS before nephrectomy. A piece of renal cortical tissue was fixed in 10% buffered formaldehyde and embedded in paraffin. Two-micrometer sections were stained with Masson trichrome. All sections were coded and analyzed in a blinded manner by two individuals, including a pathologist. The mean of both scores was used for further analysis.

The severity of glomerular extracellular matrix expansion was quantitated based on the glomerular matrix score using a previously published method [41]. Briefly, the glomerular matrix score was measured by mean score of 30 glomeruli cut at almost full diameter based on the percentage of glomerular area occupied by the extracellular matrix and hyalinosis as follows: 0 = no lesion; 1 = <10%; 2 = 10–25%; 3 = 25–50%; and 4 = >50%. The extent of IF was scored at a 250× magnification using a previously published method [41]. Briefly, the IF score was determined by the mean score of 20 cortical areas based on the percentage of areas with fibrosis as follows: 0 = no lesion; 1 = <25%; 2 = 25–50%; and 3 = >50%.

### 2.6. Metabolomic Analysis

Urine samples were thawed on ice and 100 µL of rat urine was added to 200 µL of chilled acetonitrile. After vortexing for 10 min, the mixture was centrifuged at 13,000× *g* for 20 min at 4 °C to remove particles. The supernatant was transferred to injection vials. To obtain consistent differential variables, a pooled urine sample (QC) was prepared by mixing aliquots of individual samples. The prepared QC sample was acquired through a series of injections, and data were obtained by random injection. Then, 2 µL of the prepared sample was injected onto a reverse-phase 2.1 mm × 50 mm ACQUITY 1.7 μm BEH C18 column (Waters, Milford, MA, USA) using a Waters ultra-performance liquid chromatography (UPLC) system. The column was maintained at 35 °C using the ACQUITY UPLC system (Waters, Milford, Massachusetts, USA) and the gradient was eluted with a mobile phase of 0.1% formic acid (A) and 0.1% formic acid acetonitrile (B). From the start to 0.5 min, B was held at 5%, then linearly increased to 50% in 10 min, linearly increased to 95% in 10.75 min, and kept invariable for 12.25 min. After that, B was returned to 5% in 12.5 min and maintained for a further 2.5 min. The mass profile of both the positive and negative ion electrospray ionization mode was achieved using a Waters Xevo G2 time-of-flight mass spectrometer (TOF–MS). The metabolomics raw data were imported, deconvoluted, normalized, and reviewed using Progenesis QI (version 2.3, Nonlinear Dynamics Newcastle, UK). The most suitable QC sample with the highest similarity to all other samples was chosen as the alignment reference. The retention times of all other samples aligned to the reference with sensitivity (10 ppm) and retention time limits. The abundance of each entity was normalized for all compounds. Then, we filtered out the low-quality ions with a %CV of abundance >30 in the QC. Significant differential expression was defined as a false discovery rate (FDR) adjusted *p*-value (*q*-value) < 0.05. The FDR was obtained by adjusting the raw *p*-values of the *t*-test using the method of Benjamini and Hochberg [42]. The metabolomics data set was imported into EZinfo software (Umetrics) for multivariate analysis (pareto-scaled). Principal components analysis (PCA), an unsupervised multivariate statistical analysis, was performed to examine the intrinsic variations within a group and to assess the clustering behavior between groups. Clustering of the QC samples in the PCA was assessed to reveal the stability and reproducibility of the data generated during the analytical platform.

### 2.7. mRNA Analysis

Total RNA was extracted from rat kidney tissue with the NucleoSpin RNA/Protein kit (Macherey-Nagel, Bethlehem, PA, USA). RNA later-stored kidney tissue was disrupted by a BioMasher II disposable homogenizer (Nippi, Tokyo, Japan). First-strand complementary DNA was synthesized via the reverse transcription of 1 μg of total RNA using the GoScript Reverse Transcription Oligo(dT) mix (Promega, Madison, WI, USA). A 20-μL reaction mixture was prepared with 4 μL of GoScript reaction buffer, 2 μL of random primers, 2 μL of GoScript Enzyme mix, and 12 μL comprising 1 μg RNA and nuclease-free water. The mixture was incubated for 5 min at 25 °C followed by 60 min at 4 °C, and the reverse transcriptase was inactivated for 5 min at 95 °C. To dilute the cDNA, 20 μL of nuclease-free water was added. A 20-μL PCR reaction mixture was prepared with 10 μL of the 2X GoQaq qPCR master mix (Promega), 2 μL QuantiTect Primer Assay (Qiagen, Hilden, Germany), 6 μL of nuclease-free water, and 2 μL of cDNA. Real-time PCR was performed using a CFX Connect Real-Time PCR instrument (Bio-Rad, Hercules, CA, USA) with the following cycling conditions: initial denaturation at 95 °C for 2 min, and 45 cycles of denaturation at 95 °C for 15 s and annealing/extension at 60 °C for 30 s. *GAPDH* expression was used to normalize mRNA expression.

### 2.8. Statistical Analysis

All statistical analyses were performed using SPSS for Windows version 21 (IBM SPSS Statistics, Chicago, IL, USA). Statistical significance was considered at *p* < 0.05. Differences among groups for continuous variables were assessed using non-parametric statistics with the Mann–Whitney U test or Kruskal–Wallis test. Paired *t*-tests were used to compare the time points at Week 2 and Week 12.

## 3. Results

### 3.1. Proteinuria

**Figure 3:**
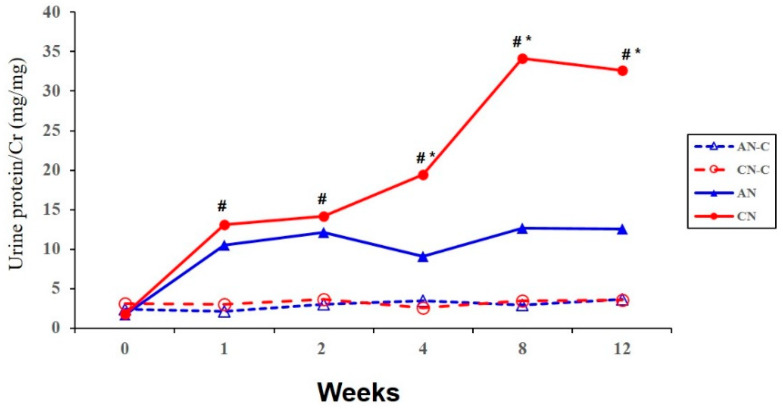
The 24-h urine protein-to-creatinine ratio in experimental glomerulonephritis rat groups. There was a marked increase in proteinuria in the CN groups compared to the AN groups from Week 4 to 12. * *p* < 0.05 vs. AN; # *p* < 0.05 vs. AN-C/CN-C. AN, acute nephritis; AN-C, control group for acute nephritis; CN, chronic nephritis; CN-C, control group for chronic nephritis.

The CN groups showed significantly different proteinuria than the CN-C or AN-C groups after one week. Moreover, there was a marked increase of proteinuria in the CN groups compared to that in the AN groups from Week 4 to Week 12 (Figure 3). These results support the hypothesis of quantitative difference of proteinuria between AN and CN groups from Week 4 to Week 12.

### 3.2. Renal Histology

**Figure 4:**
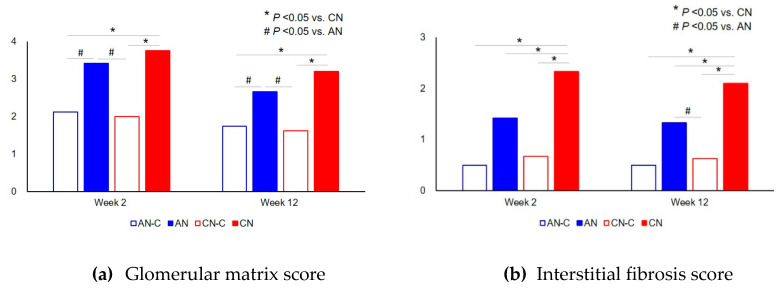
The glomerular matrix and interstitial fibrosis (IF) scores among the four groups at both the 2W and 12W time points. (**a**) glomerular matrix score, (**b**) interstitial fibrosis score. There were significant differences in both the glomerular matrix and IF scores among the four groups at both the 2W and 12W time points. AN, acute nephritis; AN-C, control group for acute nephritis; CN, chronic nephritis; CN-C, control group for chronic nephritis. * *p* < 0.05 vs. CN, # *p* < 0.05 vs. AN.

**Figure 5:**
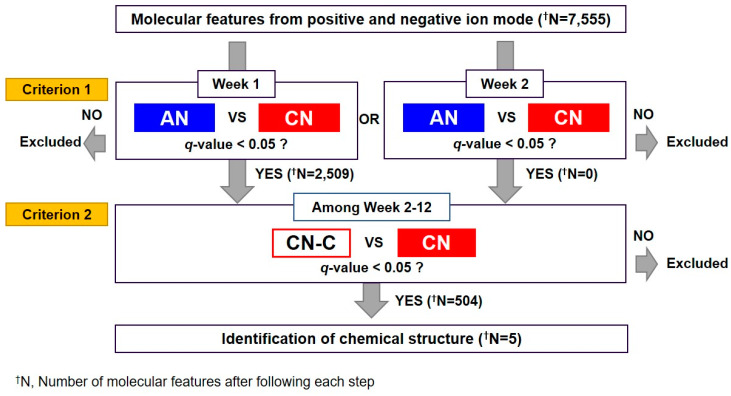
Scheme of the untargeted metabolomics strategy to determine urinary biomarkers predictive of the progression of nephritis. The predictive criterion were as follows: (**1**) *q*-value < 0.05 when the CN group was compared to the AN animal at Week 1 or 2, representing early-stage nephritis, even if there was no significant difference in proteinuria between the AN and CN groups; and (**2**) *q*-value < 0.05 when the CN group was compared to the CN-C animal between Week 2 and 12. AN, acute nephritis; AN-C, control group for acute nephritis; CN, chronic nephritis; CN-C, control group for chronic nephritis.

Figure 4 shows the glomerular matrix and IF scores among the four groups at both the 2W and 12W time points. The Kruskal–Wallis test showed a significant difference of both the glomerular matrix and IF scores among the four groups in both time points (*p* < 0.05). The glomerular matrix score of the CN group was significantly higher than that of the CN-C and AN-C groups at Weeks 2 (*p* = 0.016 and *p* = 0.009, respectively) and 12 (*p* = 0.011 and *p* = 0.017, respectively). The glomerular matrix score of the AN group was significantly higher than that of the AN-C and CN-C groups at Weeks 2 (*p* = 0.017 and *p* = 0.026, respectively) and 12 (*p* = 0.047 and *p* = 0.012, respectively). The IF score of the CN group was significantly higher than that of the AN-C, CN-C, and AN groups at both time points (*p* < 0.05). However, the IF score of the AN group was not significantly different from that of the AN-C or CN-C group at both time points. In other words, the pathological features of the AN and CN groups were significantly determined by comparisons with the AN-C and CN-C groups at Week 2, respectively. Moreover, the IF score of the CN group was persistently distinct for up to 12 weeks, unlike that of the AN group. Based on Figure 3 and Figure 4, we hypothesized that metabolic substances should differ between the AN and CN groups from one week to two weeks before the pathologic finding was confirmed. Moreover, these should also have been clearly different from those of the CN-C group from Week 2 to 12 (Figure 5).

### 3.3. Metabolomics Analysis

**Figure 6:**
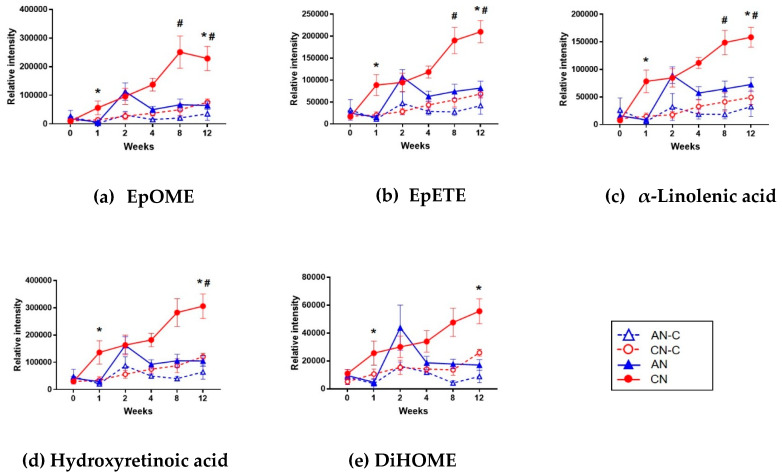
Relative intensity of the five identified urinary biomarkers predictive of the progression of nephritis. (**a**) EpOME, (**b**) EpETE, (**c**) α-linolenic acid, (**d**) hydroxyretinoic acid, and (**e**) DiHOME. Data are shown as mean values ± SEM. * *q*  < 0.05 vs. AN; # *q*  < 0.05 vs. CN-C. AN, acute nephritis; AN-C, control group for acute nephritis; CN, chronic nephritis; CN-C, control group for chronic nephritis; DiHOME, dihydroxyoctadecenoic acid; EpETE, epoxyeicosatetraenoic acid; EpOME, epoxyoctadecenoic acid.

Urine samples from AN-C, CN-C, AN, and CN were analyzed by UPLC–QTOF operated in positive and negative ionization modes (Figure S1). Figure 6 shows the selection procedure of biomarkers to predict the progression of nephritis. A total of 7555 metabolic features were detected in positive and negative modes using Progenesis QI. Unsupervised PCA analysis showed a difference between the AN and CN groups over time (Figure S1). In chronological order, the CN group proceeds in one direction, while the AN group returns in the direction of Week 0. The QC samples clustered in the middle of the PCA plot, confirming the stability and the reproducibility of the data obtained in both the positive and negative modes. We then selected metabolites that satisfied the following criteria in Figure 5: (1) *q*-value < 0.05 when the CN group was compared to the AN group at Week 1 or 2, representing early-stage nephritis, even if there was no significant difference in proteinuria between the AN and CN groups; and (2) *q*-value < 0.05 when the CN group was compared to the CN-C group between Week 2 and 12. In total, 504 metabolites met the marker selection criteria (Table S1). Five metabolites were identified as epoxyoctadecenoic acid (EpOME), epoxyeicosatetraenoic acid (EpETE), α-linolenic acid (ALA), hydroxyretinoic acid, and dihydroxyoctadecenoic acid (DiHOME) by comparing MS/MS spectra with those of the authentic compounds (Figure 6). The metabolite of EpOME, dihydroxyoctadecenoic acid (DiHOME), was further identified. DiHOME met Criterion 1, but the *q*-value was 0.0674 when the CN group was compared to the CN-C group at Week 8.

### 3.4. Renal mRNA Expression of CYP2J4, CYP2C23, CYP2E1, Ephx2, and Ephx3.

**Figure 7:**
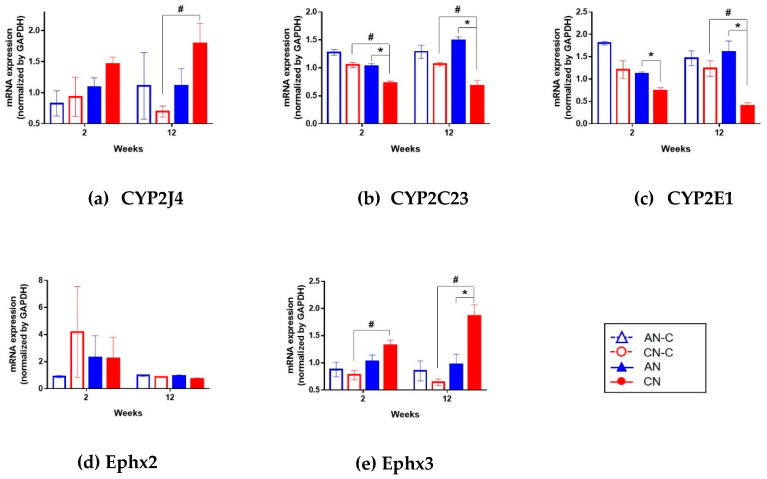
Renal mRNA expression of (**a**) *CYP2J4*, (**b**) *CYP2C23*, (**c**) *CYP2E1*, (**d**) *Ephx2*, and (**e**) *Ephx3* at the Week 2 and 12 time points. *CYP2C23* and *CYP2E1* were lower in the CN group than in AN or CN-C animals. Ephx3 expression was higher with CN than in AN or CN-C groups. The Mann–Whitney U test was used to calculate statistical significance. * *p* < 0.05 vs. AN; # *p* < 0.05 vs. CN-C. AN, acute nephritis; AN-C, control group for acute nephritis; CN, chronic nephritis; CN-C, control group for chronic nephritis; Ephx2, epoxide hydrolase 2; Ephx3, epoxide hydrolase 3.

The production of the epoxide metabolites from PUFAs has been attributed to members of the cytochrome (CYP) 2C [43], CYP2E [44], and CYP2J [45,46] families, and subsequent hydroxylation to generate their corresponding diols is catalyzed by epoxide hydrolase 2 (Ephx2) and epoxide hydrolase 3 (Ephx3) [47]. To confirm the involvement of CYP2 and Ephx in metabolic changes to PUFA metabolism, renal mRNA expression of *CYP2* and *Ephx* was evaluated. *CYP2J4* expression was higher in the CN group than the CN-C group (*p* < 0.05) at Week 12, whereas *CYP2C23* and *CYP2E1* were lower in the CN group than in the AN or CN-C group. *Ephx3* expression was higher in the CN group than in the AN or CN-C group, whereas no differences in *Ephx2* expression were observed (Figure 7).

## 4. Discussion

**Figure 8:**
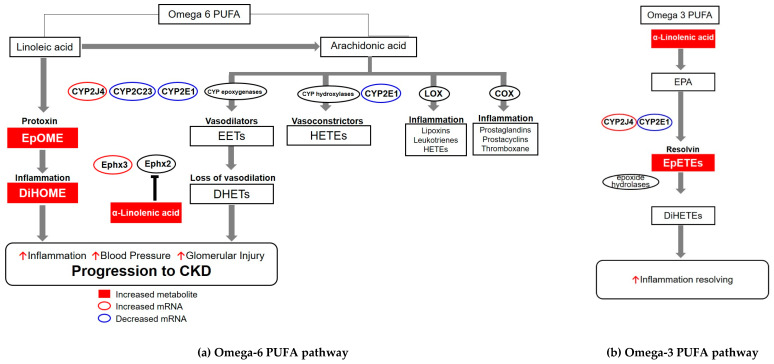
Potential mechanism of cytochrome P450 (CYP450)-dependent metabolism of polyunsaturated omega-6 and omega-3 fatty acids involved in CKD progression. (**a**) omega-6 PUFA pathway, (**b**) omega-3 PUFA pathway. Decreased mRNA expression of *CYP2C23* and *CYP2E1* prevent the production of EETs, as a vasodilator, from arachidonic acid. Increased mRNA expression of *CYP2J4* induces the generation of EpOME and the corresponding DiHOME as a protoxin via the soluble epoxide hydrolase, leading to the progression of CKD. Metabolites in the red colored squares are increased. mRNA is increased in the red line circle and decreased in the blue line circle. CKD, chronic kidney disease; COX, cyclooxygenase; DHET, dihydroxyeicosatrienoic acid; DiHETE, dihydroxyeicosatetraenoic acid; DiHOME, dihydroxyoctadecenoic acid; EETs, epoxyeicosatrienoic acids; EPA, eicosapentaenoic acid; *Ephx2*, epoxide hydrolase 2; *Ephx3*, epoxide hydrolase 3; EpOME, epoxyoctadecenoic acid; EpETE, epoxyeicosatetraenoic acid; HETE, hydroxyeicosatetraenoic acid; LOX, lipoxygenase; PUFA, polyunsaturated fatty acid.

This study investigated the differences in the pathogenesis of acute and chronic nephritis and identify urinary metabolites predicting the progression or regression of kidney injuries through an untargeted metabolomics approach, using an experimental anti-Thy1.1-initiated model of mesangioproliferative glomerulonephritis with or without heminephrectomy in rats. Figure 8 shows the metabolic pathway, affected by CYP2J4 and Ephx3 changes, on CKD progression. Decreased mRNA expression of *CYP2C23* and *CYP2E1* prevents the production of epoxyeicosatrienoic acids (EETs) as a vasodilator derived from arachidonic acid (AA). Increased mRNA expression of *CYP2J4* induces the generation of EpOME as a protoxin, which is metabolized to DiHOME and involved in inflammation via the upregulation of Ephx3 as a soluble epoxide hydrolase (sEH). These are thought to cause IF in the CN group unlike the AN group, leading to the CKD progression.

Proteinuria is the abnormal transglomerular passage of urine proteins owing to increased permeability of the glomerular basement membrane and their subsequent impaired tubular reabsorption in the proximal tubule [48]. In healthy individuals, the urinary protein content ranges between 0 and 0.1 g/m^2^/d. Heavy proteinuria exceeds 1 g/m^2^/day as in primary glomerular disease and CKD. Proteinuria may reflect a specific pathophysiological condition in the kidney resulting in damage to the glomerular filtration barrier in diseases that cause the glomerular or renal tubular injuries. Therefore, metabolomics approaches have actively been used in studies on kidney diseases [49].

Herein, changes were observed in metabolite levels associated with PUFA metabolism. Conversion of PUFAs to bioactive lipid mediators via CYP isoforms is associated with cardiovascular function [50]. ALA and linoleic acid (LA) are omega-3 and omega-6 PUFAs, respectively, and are essential fatty acids in food. Certain CYP 450 enzymes, the CYP epoxygenases, metabolize LA to EpOME, which is further metabolized to DiHOME by the sEH. DiHOME contributes to the detrimental effects of ischemia–reperfusion injury with the massive production of reactive oxygen species in isolated mouse hearts [51]. EpOME is considered a leukotoxin or protoxin because it causes significant adverse cardiac effects in animal models in the presence of sEH [52,53,54]. In previous animal studies, the pharmacological inhibition of sEH activity reduced pro-inflammatory eicosanoid levels, leading to a reduction in blood pressure [55,56,57]. Furthermore, LA can also be converted to arachidonic acid (AA), which is converted to pro-inflammatory eicosanoids, including prostaglandin E2, leukotriene B4, lipoxins, and hydroxyeicosatetraenoic acid (HETE) by cyclooxygenase (COX), lipoxygenase (LOX), and CYP hydroxylases. These pro-inflammatory eicosanoids result in cardiac dysfunction, thus promoting inflammation, vasoconstriction, and apoptosis [58].

ALA is converted to the long-chain omega-3 PUFA, eicosapentaenoic acid (EPA), or docosahexaenoic acid (DHA) [59]. A meta-analysis of observational studies reported that higher ALA intake leads to cardioprotective effects [60]. Moreover, higher EPA and DHA intake also protects against cardiovascular disease [61]. These omega-3 PUFAs are converted to eicosanoids by COX, LOX, and CYP 450 enzymes. Eicosanoids derived from omega-3 PUFAs include resolvins or protectins, which play an anti-inflammatory role [61]. Stephanie et al. reported that the anti-hypertensive effects of flaxseed ingestion are induced via the ALA-mediated inhibition of sEH in patients with hypertension [62]. In contrast, ALA levels in patients with nephrotic syndrome were higher than those in the control groups, suggesting that nephrotic syndrome potentially disrupts PUFA metabolism [63]. Significant dyslipidemia occurs in nephrotic syndrome because albuminuria accelerates a compensatory increase in hepatic lipoprotein synthesis [64]. Furthermore, dyslipidemia has renal lipotoxicity indirectly through systemic inflammation and oxidative stress [65,66,67]. Therefore, higher levels of ALA in the CN groups could be triggered through proteinuria leading to further renal histologic injury through the disturbance of PUFA metabolism.

Further, AA can be also transformed into EETs by CYP epoxygenases. EET promotes vasodilation, angiogenesis, and thrombolysis, as well as inhibits inflammation and apoptosis, which can preserve cardiac function [58]. Renal epithelial cells are one of the major sites for the production of EETs, the substrate of AA and CYP epoxygenase [68]. EETs play a role in vasodilation to increase blood flow to organs, leading to decreased peripheral vascular resistance through the inhibition of epithelial sodium channels in the kidney. The association between vascular inflammation and hypertensive renal injury based on a transgenic mouse model of sEH demonstrated the associated pathogenesis, in which increased sEH activity or decreased levels of EETs contribute to hypertension and CKD [69,70,71]. The association between diols, via CYP epoxygenases, and chronic histology in the current study is consistent with the hypothesis presented.

Epoxyeicosatetraenoic acids (EpETEs) are metabolized from EPA, omega 3 fatty acid by CYP epoxygenase. The activation of EET-forming CYP epoxygenases is known to be determined by the enzyme substrate preference and the endogenous omega 3/omega 6 PUFA ratio. The differences in preference of CYP epoxygenases often induce the metabolization of EPA to EpETEs at rates exceeding their rates in metabolizing AA to EETs [72]. Increased EpETEs are considered a metabolite of the EET-inactivating pathway from AA, owing to the opposite activation of CYP2E1 and CYP2J2 in our study.

The CYP 2 family comprises the predominant epoxygenase isoform abundantly expressed in the endothelium, myocardium, and kidney of humans. Numerous studies have demonstrated the cardioprotective effects of CYP epoxygenases and EETs, including vasodilation, antihypertensive, proangiogenic, anti-atherosclerotic, and cardioprotective effects [68,73]. The role of the CYP2 family and its metabolites in inflammation and cancer have recently attracted increasing attention. CYP2C23 is expressed as an AA epoxygenase in rat kidneys [74]. Increased vascular tone caused by the down-regulation of CYP2C23 and decreased levels of EETs, as a vasodilator, were identified in androgen-induced hypertension [75]. Moreover, high potassium intake induces AA and 11,12-EET inhibition in the epithelial sodium channel in the cortical collecting duct by increasing CYP2C23 activity and decreasing sEH activity, respectively [76]. In our study, CYP2C23 expression was suppressed in CN animals compared to that in the AN group in Figure 7. This suggests that CYP2C23 downregulation is associated with renal disease progression.

CYP2E1 is known to act as a monooxygenase to metabolize AA to 19-HETE and as an epoxygenase to metabolize EPA to EpETEs [44,77]. In our study, decreased mRNA expression of *CYP2E1* was confirmed in the CN group compared to that in AN. This suggests that, as with CYP2C23, the downregulation of CYP2E1 activity induce the production of EpOME, leading to the progression of CKD.

CYP2J4 in rats is an ortholog of human CYP2J2 and mouse CYP2J6 [78]. Human CYP2J2 epoxygenase metabolizes AA to EETs and EPA to EpETEs [79]. However, this situation is complicated because although cardioprotective effects producing EETs and EpETEs were observed in young mice, protective effects were lost in aged mice with the cardiomyocyte-specific overexpression of CYP2J2, which might be attributed to increased levels of DiHOME as a leukotoxin diol [53]. In our study, CYP2J4 expression was increased in the CN group compared to that in CN-C animals. The difference in CYP2J4 between the AN and CN groups was not significant. Therefore, the activation of CYP2J4 was thought to be related to aging.

Ephx2 is a member of the epoxide hydrolase family as a sEH in humans. Ephx2 is upregulated in the liver and also in the renal proximal tubule [80]. The well-known role of Ephx2 is the regulation of hypertension in the kidney. In the presence of Ephx2, EETs as a vasodilator are rapidly metabolized into a less inactive molecule of dihydroxyeicosatrienoic acids (DHETs), leading to the elimination of the vasodilatory signal [81]. Our results suggest that the Ephx2 pathway of EET metabolism from AA is not activated.

Ephx3 is the third identified isozyme in a set of epoxide hydrolases. Ephx3 exhibits epoxide hydrolase activity with a substrate preference for 9,10-EpOME and 11,12-EET in vitro [47]. However, its endogenous function is not well known. In our study, the progression of renal disease was consistent with the association between CN and activated Ephx3, leading to an increase in EpOME as a protoxin and an increase in its metabolite, DiHOME.

The cardioprotective effects of sEH inhibitors are gradually expanding therapeutically toward renal diseases, including hypertension, diabetic nephropathy, drug-induced nephrotoxicity, and renal fibrotic disease [73]. Renal fibrosis is prevented by a sEH inhibitor in a mouse model of unilateral ureteral obstruction [82]. Diabetic nephropathy is also attenuated by sEH inhibitors, which have anti-diabetic and anti-inflammatory effects [83]. Based on this, omega-3 PUFA is emerging as an alternative for the treatment of IgA nephropathy [84]. Moreover, it has been reported that fish oil can be used to treat patients with Henoch–Schönlein purpura [85,86]. Further studies are required to investigate whether inhibition of sEH or PUFA prevents renal interstitial fibrosis and inflammation.

Retinal is converted to their corresponding retinols, in which the reverse reaction is possible with alcohol dehydrogenases. Retinal is further oxidized to retinoic acid, which promotes cell differentiation. Retinoic acid is metabolized by CYP hydroxylase to form hydroxyretinoic acid, thus diminishing the effect of cell differentiation [87].

In humans, conjugated LA and the related conjugated linolenic acids are synthesized from LA and ALA, respectively, by ruminal bacteria. Conjugated LAs are obtained from the diet [88]. While conjugated LAs increase tissue levels of retinol (vitamin A), the precise interactions between vitamin A and conjugated LA in kidney disease have remained unclear [89]. However, combined up-regulated retinol metabolism and PUFA metabolism were observed in catalase-knockout mice fed on a high-fat diet, thus contributing to liver inflammation [90]. Further studies are required to determine whether dysregulation of retinol metabolism and PUFA metabolism promote chronic inflammation in the kidneys.

One of the limitations was the results of the metabolic analysis obtained with plasma before urine testing. The plasma concentration of the metabolites was very low and therefore the plasma metabolites could not be compared with the urine metabolites. In other words, our findings indicate that metabolites in urine are associated with nephritis, independent of those in the plasma. However, further studies are required in this regard to validate our findings.

## 5. Conclusions

This article is noteworthy because it is an experimental study comparing acute and chronic nephritis to investigate the mechanism of disease progression. Our results suggest that the progression of renal disease is associated with abnormally activated epoxide hydrolase and CYP450 in the metabolism of omega-6 PUFA, leading to an increase in EpOME as pro-inflammatory eicosanoids based on the metabolomic analysis using experimental mesangial proliferative glomerulonephritis with or without heminephrectomy. Further study is necessary to estimate the causal relationship between the conversion of PUFA to pro-inflammatory eicosanoids by the epoxide hydrolase and CYP450 in terms of CKD and disease progression in humans with reversible and irreversible mesangial proliferative glomerulonephritis.

## Supplementary Materials

The following are available online at https://www.mdpi.com/2218-1989/10/4/169/s1, Figure S1: PCA score plots comparing the AN-C, AN, CN-C, and CN groups from Week 0 to 12 derived from untargeted metabolomics analysis: (**a**) positive ion mode and (**b**) negative ion mode, Table S1: List of selected metabolites that satisfied the following criteria at *q*-value < 0.05: 1) when the CN group was compared to the AN animals at Week 1 or 2; and 2) when the CN group was compared to the CN-C or AN animals between Week 4 and 12.

## Abbreviations

**AA**	**Arachidonic Acid**
AAALAC International	Association for Assessment and Accreditation of Laboratory Animal Care International
AKI	acute kidney injury
ALA	α-linolenic acid
AN	acute nephritis
CKD	chronic kidney disease
CN	chronic nephritis
COX	cyclooxygenase
CYP	cytochrome
DHA	docosahexaenoic acid
DHET	dihydroxyeicosatrienoic acid
DiHETE	dihydroxyeicosatetraenoic acid
DiHOME	dihydroxyoctadecenoic acid
EET	epoxyeicosatrienoic acid
EPA	eicosapentaenoic acid
EpETE	epoxyeicosatetraenoic acid
Ephx2	epoxide hydrolase 2
Ephx3	epoxide hydrolase 3
EpOME	epoxyoctadecenoic acid
FDR	false discovery rate
HETE	hydroxyeicosatetraenoic acid
LA	linoleic acid
LOX	lipoxygenase
PBS	phosphate-buffered saline
PCA	principal components analysis
PUFA	polyunsaturated fatty acid
sEH	soluble epoxide hydrolase
TOF/MS	time-of-flight mass spectrometer
UPLC	ultra-performance liquid chromatography

## References

[1] Hakemi M.S. (2014). Chronic kidney disease epidemiology. Iran J. Kidney Dis..

[2] Lopez-Giacoman S., Madero M. (2015). Biomarkers in chronic kidney disease, from kidney function to kidney damage. World J. Nephrol..

[3] Nkuipou-Kenfack E., Duranton F., Gayrard N., Argilés À., Lundin U., Weinberger K.M., Dakna M., Delles C., Mullen W., Husi H. (2014). Assessment of metabolomic and proteomic biomarkers in detection and prognosis of progression of renal function in chronic kidney disease. PLoS ONE.

[4] Edwards K.D., Whyte H.M. (1959). Plasma creatinine level and creatinine clearance as tests of renal function. Australas. Ann. Med..

[5] Cockcroft D.W., Gault M.H. (1976). Prediction of creatinine clearance from serum creatinine. Nephron.

[6] Hojs R., Bevc S., Ekart R., Gorenjak M., Puklavec L. (2008). Serum cystatin C as an endogenous marker of renal function in patients with chronic kidney disease. Ren. Fail..

[7] Friedman A., Avner E., Harmon W., Niaudet P., Yoshikawa N. (2009). Laboratory assessment and investigation of renal function. Pediatric Nephrology.

[8] American Society of Nephrology (2005). American society of nephrology renal research report. J. Am. Soc. Nephrol..

[9] Herget-Rosenthal S., Marggraf G., Hüsing J., Göring F., Pietruck F., Janssen O., Philipp T., Kribben A. (2004). Early detection of acute renal failure by serum cystatin C. Kidney Int..

[10] Parikh C.R., Abraham E., Ancukiewicz M., Edelstein C.L. (2005). Urine IL-18 is an early diagnostic marker for acute kidney injury and predicts mortality in the intensive care unit. J. Am. Soc. Nephrol..

[11] Mishra J., Dent C., Tarabishi R., Mitsnefes M.M., Ma Q., Kelly C., Ruff S.M., Zahedi K., Shao M., Bean J. (2005). Neutrophil gelatinase-associated lipocalin (NGAL) as a biomarker for acute renal injury after cardiac surgery. Lancet.

[12] Coca S.G., Yalavarthy R., Concato J., Parikh C.R. (2008). Biomarkers for the diagnosis and risk stratification of acute kidney injury: A systematic review. Kidney Int..

[13] Fan L., Zhang W., Yin M., Zhang T., Wu X., Zhang H., Sun M., Li Z., Hou Y., Zhou X. (2012). Identification of metabolic biomarkers to diagnose epithelial ovarian cancer using a UPLC/QTOF/MS platform. Acta Oncol..

[14] Posada-Ayala M., Zubiri I., Martin-Lorenzo M., Sanz-Maroto A., Molero D., Gonzalez-Calero L., Fernandez-Fernandez B., de la Cuesta F., Laborde C.M., Barderas M.G. (2014). Identification of a urine metabolomic signature in patients with advanced-stage chronic kidney disease. Kidney Int..

[15] Gao Y. (2015). Urine is a better biomarker source than blood especially for kidney diseases. Adv. Exp. Med. Biol..

[16] Jing J., Gao Y. (2018). Urine biomarkers in the early stages of diseases: Current status and perspective. Discov. Med..

[17] Hwang G.S., Yang J.Y., Ryu D.H., Kwon T.H. (2010). Metabolic profiling of kidney and urine in rats with lithium-induced nephrogenic diabetes insipidus by (1)H-NMR-based metabonomics. Am. J. Physiol. Renal Physiol..

[18] Zivkovic A.M., Yang J., Georgi K., Hegedus C., Nording M.L., O’Sullivan A., German J.B., Hogg R.J., Weiss R.H., Bay C. (2012). Serum oxylipin profiles in IgA nephropathy patients reflect kidney functional alterations. Metabolomics.

[19] Chen D.Q., Cao G., Chen H., Argyopoulos C.P., Yu H., Su W., Chen L., Samuels D.C., Zhuang S., Bayliss G.P. (2019). Identification of serum metabolites associating with chronic kidney disease progression and anti-fibrotic effect of 5-methoxytryptophan. Nat. Commun..

[20] Zhao Y.Y., Cheng X.L., Wei F., Bai X., Lin R.C. (2012). Application of faecal metabonomics on an experimental model of tubulointerstitial fibrosis by ultra performance liquid chromatography/high-sensitivity mass spectrometry with MSE data collection technique. Biomarkers.

[21] Zhao Y.Y., Shen X., Cheng X.L., Wei F., Bai X., Lin R.C. (2012). Urinary metabonomics study on the protective effects of ergosta-4,6,8(14),22-tetraen-3-one on chronic renal failure in rats using UPLC Q-TOF/MS and a novel MS^E^ data collection technique. Process. Biochem..

[22] Zhang Z.H., Wei F., Vaziri N.D., Cheng X.L., Bai X., Lin R.C., Zhao Y.Y. (2015). Metabolomics insights into chronic kidney disease and modulatory effect of rhubarb against tubulointerstitial fibrosis. Sci. Rep..

[23] Zhao Y.Y., Wang H.L., Cheng X.L., Wei F., Bai X., Lin R.C., Vaziri N.D. (2015). Metabolomics analysis reveals the association between lipid abnormalities and oxidative stress, inflammation, fibrosis, and Nrf2 dysfunction in aristolochic acid-induced nephropathy. Sci. Rep..

[24] Fang J., Wang W., Sun S., Wang Y., Li Q., Lu X., Hao Z., Zhang Y. (2015). A urine metabonomics study of chronic renal failure and intervention effects of total aglycone extracts of *Scutellaria baicalensis* in 5/6 nephrectomy rats. RSC Adv..

[25] Nagata M., Avner E., Harmon W., Niaudet P., Yoshikawa N. (2009). Immune-mediated glomerular injury. Pediatric Nephrology.

[26] Sakai N., Iseki K., Suzuki S., Mori T., Hagino S., Zhang Y., Yokoya S., Kawasaki Y., Suzuki J., Isome M. (2005). Uninephrectomy induces progressive glomerulosclerosis and apoptosis in anti-Thy1 glomerulonephritis. Pathol. Int..

[27] Kobayashi Y., Tateno S., Hiki Y., Shigematsu H. (1983). IgA nephropathy: Prognostic significance of proteinuria and histological alterations. Nephron.

[28] Reich H.N., Troyanov S., Scholey J.W., Cattran D.C. (2007). Remission of proteinuria improves prognosis in IgA nephropathy. J. Am. Soc. Nephrol..

[29] Hara M., Yanagihara T., Kihara I. (2007). Cumulative excretion of urinary podocytes reflects disease progression in IgA nephropathy and Schönlein-Henoch purpura nephritis. Clin. J. Am. Soc. Nephrol..

[30] Becherucci F., Roperto R.M., Materassi M., Romagnani P. (2016). Chronic kidney disease in children. Clin. Kidney J..

[31] Kellum J.A., Lameire N. (2018). The definition of acute kidney injury. Lancet.

[32] National Research Council, Division on Earth and Life Studies, Institute for Laboratory Animal Research, Committee for the Update of the Guide for the Care and Use of Laboratory Animals (2011). Environment, housing, and management. Guide for the Care and Use of Laboratory Animals.

[33] Steinmann-Niggli K., Ziswiler R., Küng M., Marti H.P. (1998). Inhibition of matrix metalloproteinases attenuates anti-Thy1.1 nephritis. J. Am. Soc. Nephrol..

[34] Aben J.A., IJpelaar D.H., Baelde H., Worley P., Noble N., Bruijn J.A., de Heer E. (2006). Glomerular expression of neuronal activity-regulated pentraxin precedes the development of anti-Thy-1-induced progressive glomerulosclerosis. Kidney Int..

[35] Cheng Q.L., Orikasa M., Morioka T., Kawachi H., Chen X.M., Oite T., Shimizu F. (1995). Progressive renal lesions induced by administration of monoclonal antibody 1-22-3 to unilaterally nephrectomized rats. Clin. Exp. Immunol..

[36] Seegal B.C., Loeb E.N. (1946). The production of chronic glomerulonephritis in rats by the injection of rabbit anti-rat-placenta serum. J. Exp. Med..

[37] Schaefer L., Hausser H., Altenburger M., Ugorcakova J., August C., Fisher L.W., Schaefer R.M., Kresse H. (1998). Decorin, biglycan and their endocytosis receptor in rat renal cortex. Kidney Int..

[38] Terashima H., Kato M., Ebisawa M., Kobayashi H., Suzuki K., Nezu Y., Sada T. (2014). R-268712, an orally active transforming growth factor-β type I receptor inhibitor, prevents glomerular sclerosis in a Thy1 nephritis model. Eur. J. Pharmacol..

[39] Larsen K. (1972). Creatinine assay by a reaction-kinetic principle. Clin. Chim. Acta.

[40] Okuda S., Languino L.R., Ruoslahti E., Border W.A. (1990). Elevated expression of transforming growth factor-beta and proteoglycan production in experimental glomerulonephritis. Possible role in expansion of the mesangial extracellular matrix. J. Clin. Invest.

[41] Reinhard M.K., Hottendorf G.H., Powell E.D. (1991). Differences in the sensitivity of Fischer and Sprague-Dawley rats to aminoglycoside nephrotoxicity. Toxicol. Pathol..

[42] Benjamini Y., Hochberg Y. (1995). Controlling the false discovery rate: A practical and powerful approach to multiple testing. J. R. Stat. Soc. Ser. B Methodol..

[43] Karara A., Makita K., Jacobson H.R., Falck J.R., Guengerich F.P., DuBois R.N., Capdevila J.H. (1993). Molecular cloning, expression, and enzymatic characterization of the rat kidney cytochrome P-450 arachidonic acid epoxygenase. J. Biol. Chem..

[44] Laethem R.M., Balazy M., Falck J.R., Laethem C.L., Koop D.R. (1993). Formation of 19 (S)-, 19 (R)-, and 18 (R)-hydroxyeicosatetraenoic acids by alcohol-inducible cytochrome P450 2E1. J. Biol. Chem..

[45] Yu Z., Huse L.M., Adler P., Graham L., Ma J., Zeldin D.C., Kroetz D.L. (2000). Increased CYP2J expression and epoxyeicosatrienoic acid formation in spontaneously hypertensive rat kidney. Mol. Pharmacol..

[46] Zhang Q.Y., Ding X., Kaminsky L.S. (1997). cDNA cloning, heterologous expression, and characterization of rat intestinal CYP2J4. Arch. Biochem. Biophys..

[47] Hoopes S.L., Gruzdev A., Edin M.L., Graves J.P., Bradbury J.A., Flake G.P., Lih F.B., DeGraff L.M., Zeldin D.C. (2017). Generation and characterization of epoxide hydrolase 3 (EPHX3)-deficient mice. PLoS ONE.

[48] D’Amico G., Bazzi C. (2003). Pathophysiology of proteinuria. Kidney Int..

[49] Dubin R.F., Rhee E.P. (2020). Proteomics and metabolomics in kidney disease, including insights into etiology, treatment, and rrevention. Clin. J. Am. Soc. Nephrol..

[50] Fleming I. (2014). The pharmacology of the cytochrome P450 epoxygenase/soluble epoxide hydrolase axis in the vasculature and cardiovascular disease. Pharmacol. Rev..

[51] Edin M.L., Wang Z., Bradbury J.A., Graves J.P., Lih F.B., DeGraff L.M., Foley J.F., Torphy R., Ronnekleiv O.K., Tomer K.B. (2011). Endothelial expression of human cytochrome P450 epoxygenase CYP2C8 increases susceptibility to ischemia-reperfusion injury in isolated mouse heart. FASEB J..

[52] Spector A.A., Kim H.Y. (2015). Cytochrome P450 epoxygenase pathway of polyunsaturated fatty acid metabolism. Biochim. Biophys. Acta.

[53] Chaudhary K.R., Zordoky B.N., Edin M.L., Alsaleh N., El-Kadi A.O., Zeldin D.C., Seubert J.M. (2013). Differential effects of soluble epoxide hydrolase inhibition and CYP2J2 overexpression on postischemic cardiac function in aged mice. Prostaglandins Other Lipid Mediat..

[54] Stimers J.R., Dobretsov M., Hastings S.L., Jude A.R., Grant D.F. (1999). Effects of linoleic acid metabolites on electrical activity in adult rat ventricular myocytes. Biochim. Biophys. Acta.

[55] Honetschlägerová Z., Kitada K., Husková Z., Sporková A., Kopkan L., Bürgelová M., Varcabová Š., Nishiyama A., Hwang S.H., Hammock B.D. (2013). Antihypertensive and renoprotective actions of soluble epoxide hydrolase inhibition in ANG II-dependent malignant hypertension are abolished by pretreatment with L-NAME. J. Hypertens..

[56] Neckář J., Kopkan L., Husková Z., Kolář F., Papoušek F., Kramer H.J., Hwang S.H., Hammock B.D., Imig J.D., Malý J. (2012). Inhibition of soluble epoxide hydrolase by cis-4-[4-(3-adamantan-1-ylureido)cyclohexyl-oxy] benzoic acid exhibits antihypertensive and cardioprotective actions in transgenic rats with angiotensin II-dependent hypertension. Clin. Sci..

[57] Imig J.D., Zhao X., Zaharis C.Z., Olearczyk J.J., Pollock D.M., Newman J.W., Kim I.H., Watanabe T., Hammock B.D. (2005). An orally active epoxide hydrolase inhibitor lowers blood pressure and provides renal protection in salt-sensitive hypertension. Hypertension.

[58] Jamieson K.L., Endo T., Darwesh A.M., Samokhvalov V., Seubert J.M. (2017). Cytochrome P450-derived eicosanoids and heart function. Pharmacol. Ther..

[59] Davidson M.H. (2013). Omega-3 fatty acids: New insights into the pharmacology and biology of docosahexaenoic acid, docosapentaenoic acid, and eicosapentaenoic acid. Curr. Opin. Lipidol..

[60] Pan A., Chen M., Chowdhury R., Wu J.H., Sun Q., Campos H., Mozaffarian D., Hu F.B. (2012). α -Linolenic acid and risk of cardiovascular disease: A systematic review and meta-analysis. Am. J. Clin. Nutr..

[61] Mozaffarian D., Wu J.H. (2011). Omega-3 fatty acids and cardiovascular disease: Effects on risk factors, molecular pathways, and clinical events. J. Am. Coll. Cardiol..

[62] Caligiuri S.P., Aukema H.M., Ravandi A., Guzman R., Dibrov E., Pierce G.N. (2014). Flaxseed consumption reduces blood pressure in patients with hypertension by altering circulating oxylipins via an alpha-linolenic acid-induced inhibition of soluble epoxide hydrolase. Hypertension.

[63] Fujita T., Nakamura N., Kumasaka R., Shimada M., Murakami R., Osawa H., Yamabe H., Okumura K. (2006). Comparison of lipid and fatty acid metabolism between minimal change nephrotic syndrome and membranous nephropathy. In Vivo.

[64] Moorhead J.F., Chan M.K., El-Nahas M., Varghese Z. (1982). Lipid nephrotoxicity in chronic progressive glomerular and tubulo-interstitial disease. Lancet.

[65] Weinberg J.M. (2006). Lipotoxicity. Kidney Int..

[66] Ruan X.Z., Varghese Z., Moorhead J.F. (2009). An update on the lipid nephrotoxicity hypothesis. Nat. Rev. Nephrol..

[67] Wahba I.M., Mak R.H. (2007). Obesity aatcand obesity-initiated metabolic syndrome: Mechanistic links to chronic kidney disease. Clin. J. Am. Soc. Nephrol..

[68] Capdevila J., Wang W. (2013). Role of cytochrome P450 epoxygenase in regulating renal membrane transport and hypertension. Curr. Opin. Nephrol. Hypertens..

[69] Imig J.D. (2015). Epoxyeicosatrienoic acids, hypertension, and kidney injury. Hypertension.

[70] Kaergel E., Muller D.N., Honeck H., Theuer J., Shagdarsuren E., Mullally A., Luft F.C., Schunck W.H. (2002). P450-dependent arachidonic acid metabolism and angiotensin II–induced renal damage. Hypertension.

[71] Zhao X., Pollock D.M., Inscho E.W., Zeldin D.C., Imig J.D. (2003). Decreased renal cytochrome P450 2C enzymes and impaired vasodilation are associated with angiotensin salt-sensitive hypertension. Hypertension.

[72] Frömel T., Fleming I. (2015). Whatever happened to the epoxyeicosatrienoic acid-like endothelium-derived hyperpolarizing factor? The identification of novel classes of lipid mediators and their role in vascular homeostasis. Antioxid. Redox. Signal..

[73] Imig J.D. (2018). Prospective for cytochrome P450 epoxygenase cardiovascular and renal therapeutics. Pharmacol. Ther..

[74] Imaoka S., Wedlund P.J., Ogawa H., Kimura S., Gonzalez F.J., Kim H.Y. (1993). Identification of CYP2C23 expressed in rat kidney as an arachidonic acid epoxygenase. J. Pharmacol. Exp. Ther..

[75] Singh H., Schwartzman M.L. (2008). Renal vascular cytochrome P450-derived eicosanoids in androgen-induced hypertension. Pharmacol. Rep..

[76] Sun P., Lin D.H., Yue P., Jiang H., Gotlinger K.H., Schwartzman M.L., Falck J.R., Goli M., Wang W.H. (2010). High potassium intake enhances the inhibitory effect of 11,12-EET on ENaC. J. Am. Soc. Nephrol..

[77] Porubsky P.R., Meneely K.M., Scott E.E. (2008). Structures of human cytochrome P-450 2E1. Insights into the binding of inhibitors and both small molecular weight and fatty acid substrates. J. Biol. Chem..

[78] Behmoaras J., Diaz A.G., Venda L., Ko J.H., Srivastava P., Montoya A., Faull P., Webster Z., Moyon B., Pusey C.D. (2015). Macrophage epoxygenase determines a profibrotic transcriptome signature. J. Immunol..

[79] Westphal C., Konkel A., Schunck W.H. (2011). CYP-eicosanoids--a new link between omega-3 fatty acids and cardiac disease?. Prostaglandins Other Lipid Mediat..

[80] Morisseau C., Hammock B.D. (2007). Measurement of soluble epoxide hydrolase (sEH) activity. Curr. Protoc. Toxicol..

[81] Imig J.D. (2005). Epoxide hydrolase and epoxygenase metabolites as therapeutic targets for renal diseases. Am. J. Physiol. Renal Physiol..

[82] Kim J., Imig J.D., Yang J., Hammock B.D., Padanilam B.J. (2014). Inhibition of soluble epoxide hydrolase prevents renal interstitial fibrosis and inflammation. Am. J. Physiol. Renal Physiol..

[83] Elmarakby A.A., Faulkner J., Al-Shabrawey M., Wang M.H., Maddipati K.R., Imig J.D. (2011). Deletion of soluble epoxide hydrolase gene improves renal endothelial function and reduces renal inflammation and injury in streptozotocin-induced type 1 diabetes. Am. J. Physiol. Regul. Integr. Comp. Physiol..

[84] Hirahashi J. (2017). Omega-3 polyunsaturated fatty acids for the treatment of IgA nephropathy. J. Clin. Med..

[85] Barnadas M.A., Díaz Encarnación M.M. (2016). Refractory cutaneous IgA vasculitis treated with omega-3 fatty acids. Case Rep. Dermatol..

[86] Dixit M.P., Dixit N.M., Scott K. (2004). Managing Henoch-Schonlein purpura in children with fish oil and ACE inhibitor therapy. Nephrology.

[87] Rižner T.L. (2012). Enzymes of the AKR1B and AKR1C subfamilies and uterine diseases. Front. Pharmacol..

[88] McIntosh F.M., Shingfield K.J., Devillard E., Russell W.R., Wallace R.J. (2009). Mechanism of conjugated linoleic acid and vaccenic acid formation in human faecal suspensions and pure cultures of intestinal bacteria. Microbiology.

[89] Carta G., Murru E., Cordeddu L., Ortiz B., Giordano E., Belury M.A., Quadro L., Banni S. (2014). Metabolic interactions between vitamin A and conjugated linoleic acid. Nutrients.

[90] Na J., Choi S.A., Khan A., Huh J.Y., Piao L., Hwang I., Ha H., Park Y.H. (2019). Integrative omics reveals metabolic and transcriptomic alteration of nonalcoholic fatty liver disease in catalase knockout mice. Biomol. Ther..

