# Supplementary material for: Mechanism of Chronic Kidney Disease Progression and Novel Biomarkers: A Metabolomic Analysis of Experimental Glomerulonephritis
Source: Metabolites. 2020 Apr 24;10(4):169. doi: 10.3390/metabo10040169 (PMC7240957; doi:10.3390/metabo10040169)
Supplement: Supplementary file 1 [file metabolites-10-00169-s001.zip › Supplementary Table S1.pdf]

ESI	Neutral mass (Da)	Mass (m/z)	Retention time (min)	Metabolites	$q < 0.05$			
					AN vs CN at Week 1	CN-C vs CN at Week 4	CN-C vs CN at Week 8	CN-C vs CN at Week 12
POS	-	115.0378	1.08	-	x		x	x
NEG	311.9745	332.9501	1.15	-	x		x	
NEG	192.0261	191.0185	1.18	-	x		x	
NEG	130.0255	111.0076	1.18	-	x		x	
NEG	86.0360	85.0287	1.18	-	x		x	
POS	-	215.0175	1.18	-	x		x	
POS	-	216.0245	1.18	-	x		x	
POS	-	407.0327	1.18	-	x		x	
NEG	-	87.0080	1.19	-	x		x	
NEG	-	154.9979	1.19	-	x		x	
POS	-	122.0146	1.19	-	x		x	
NEG	-	249.0060	1.21	-	x		x	
POS	-	248.1543	1.21	-	x		x	
POS	-	423.0084	1.21	-	x		x	
NEG	-	263.0225	1.39	-	x		x	
POS	-	330.9951	1.40	-	x		x	
POS	-	113.0245	1.50	-	x		x	
POS	-	197.0675	1.52	-	x	x		
POS	-	174.1241	1.56	-	x		x	
NEG	-	129.0185	1.83	-	x		x	
NEG	-	222.9905	1.83	-	x		x	
NEG	225.9479	270.9496	1.85	-	x		x	
NEG	-	85.0287	1.85	-	x		x	
NEG	-	336.9210	1.88	-	x		x	
NEG	-	205.0336	2.31	-	x		x	
NEG	-	204.9807	2.51	-	x			x
POS	-	124.0760	2.82	-	x		x	
NEG	-	188.9854	3.05	-	x		x	
NEG	-	321.0391	3.16	-	x		x	x
POS	-	367.0218	3.16	-	x		x	
POS	-	158.0816	3.37	-	x			x
POS	-	120.0449	3.39	-	x		x	x
NEG	-	154.0240	4.03	-	x		x	
POS	-	127.0362	4.23	-	x		x	
NEG	-	216.9803	4.26	-	x		x	x
NEG	-	127.0756	4.30	-	x		x	
NEG	-	137.0233	4.34	-	x		x	
NEG	-	182.0212	4.44	-	x		x	x

NEG	-	197.0445	4.51	-	x		x	x
NEG	-	224.0552	4.52	-	x		x	
NEG	-	336.9649	4.52	-	x		x	
POS	-	160.0763	4.52	-	x		x	
POS	191.0947	174.0914	4.65	-	x		x	
NEG	-	249.0027	4.91	-	x		x	
POS	-	280.1510	4.98	-	x	x		
NEG	-	278.0694	5.00	-	x		x	
POS	-	280.1548	5.00	-	x	x	x	
NEG	249.0644	270.0404	5.02	-	x		x	x
POS	-	143.0404	5.02	-	x		x	
POS	-	402.1673	5.07	-	x		x	
NEG	172.1091	153.0913	5.15	-	x			x
POS	-	239.0898	5.17	-	x		x	
NEG	-	313.0715	5.33	-	x		x	
NEG	-	258.9913	5.42	-	x		x	
POS	-	229.1086	5.48	-	x		x	
POS	179.0585	162.0552	5.53	-	x	x	x	x
NEG	-	331.9513	5.59	-	x		x	
POS	-	302.1948	5.66	-	x	x		x
POS	-	319.1652	5.69	-	x	x	x	x
POS	-	151.0762	5.77	-	x		x	
POS	227.1527	210.1495	5.80	-	x		x	
POS	-	135.1167	5.80	-	x		x	
NEG	-	179.0649	5.81	-	x		x	x
POS	-	182.1283	5.87	-	x			x
POS	-	332.0920	5.88	-	x			x
POS	-	136.0416	5.90	-	x	x		
NEG	-	296.0925	5.95	-	x		x	
POS	-	213.1162	5.97	-	x		x	
POS	-	123.0812	5.97	-	x			x
POS	-	181.0874	5.97	-	x		x	
POS	-	259.1022	5.97	-	x		x	
NEG	-	281.0595	6.01	-	x		x	
POS	-	116.0711	6.03	-	x		x	
NEG	416.0846	415.0773	6.12	-	x		x	
NEG	-	295.1288	6.12	-	x		x	
NEG	-	253.1184	6.12	-	x		x	x
NEG	-	613.2490	6.12	-	x		x	
NEG	-	379.0806	6.13	-	x		x	

POS	-	381.1515	6.14	-	x		x	
POS	-	295.1134	6.14	-	x		x	
POS	-	150.0780	6.16	-	x		x	
POS	-	283.1120	6.16	-	x		x	
NEG	151.0626	150.0553	6.17	-	x		x	
NEG	210.0896	255.0878	6.31	-	x		x	
POS	-	249.1067	6.31	-	x		x	
POS	-	251.1295	6.31	-	x		x	
NEG	-	375.1302	6.32	-	x		x	
NEG	-	195.1008	6.34	-	x		x	
POS	262.0831	263.0903	6.35	-	x		x	
POS	-	284.1867	6.41	-	x		x	
POS	-	359.1827	6.41	-	x		x	
POS	-	344.2084	6.41	-	x		x	
POS	-	465.2202	6.45	-	x	x		
NEG	128.0821	127.0754	6.47	-	x	x	x	
POS	-	155.0720	6.47	-	x		x	
NEG	163.0281	184.0038	6.50	-	x		x	
NEG	-	331.0420	6.50	-	x		x	
NEG	-	155.0689	6.52	-	x		x	
NEG	163.0625	162.0547	6.53	-	x		x	
NEG	-	323.1017	6.53	-	x		x	
NEG	-	215.1283	6.61	-	x		x	
POS	-	123.0805	6.61	-	x		x	
POS	-	125.0602	6.63	-	x		x	
POS	-	284.1208	6.63	-	x		x	
NEG	205.0737	204.0665	6.65	-	x		x	
NEG	-	109.0285	6.65	-	x		x	
NEG	-	116.0496	6.65	-	x		x	
POS	-	299.0863	6.65	-	x		x	
POS	-	403.2106	6.65	-	x		x	x
NEG	-	158.0601	6.67	-	x		x	x
POS	-	329.2143	6.68	-	x		x	
POS	-	387.2140	6.68	-	x		x	
POS	-	381.1865	6.69	-	x		x	
POS	-	491.1695	6.69	-	x		x	x
NEG	-	193.0489	6.74	-	x		x	
POS	148.0883	149.0963	6.75	-	x		x	
POS	-	241.0749	6.75	-	x		x	x
POS	-	225.1104	6.75	-	x		x	

NEG	-	135.0076	6.78	-	x		x	
POS	-	323.1041	6.79	-	x		x	
POS	-	326.1077	6.83	-	x		x	
NEG	-	655.1839	6.85	-	x		x	
NEG	216.0983	237.0736	6.86	-	x		x	
POS	206.0946	207.0994	6.89	-	x		x	
POS	-	111.0807	6.89	-	x		x	
POS	-	285.1254	6.90	-	x		x	
POS	-	257.0767	6.93	-	x			x
POS	-	344.2434	6.93	-	x		x	x
POS	-	387.2084	6.97	-	x		x	x
POS	-	273.1070	6.97	-	x		x	
POS	-	387.2105	6.98	-	x		x	
POS	-	239.0612	7.01	-	x		x	
POS	-	181.0236	7.01	-	x		x	
NEG	160.1088	159.1014	7.02	-	x		x	
NEG	264.1011	263.0930	7.02	-	x		x	
POS	-	132.9971	7.02	-	x		x	
POS	-	165.0205	7.02	-	x		x	
POS	-	283.1018	7.02	-	x		x	
POS	-	190.0176	7.02	-	x		x	
NEG	146.0933	127.0755	7.04	-	x		x	
NEG	232.1269	253.1073	7.04	-	x		x	
POS	-	319.1268	7.08	-	x		x	x
POS	-	239.1266	7.11	-	x		x	
POS	-	261.1099	7.11	-	x			x
POS	-	229.0817	7.12	-	x			x
NEG	-	155.0688	7.23	-	x		x	
POS	-	368.1171	7.23	-	x		x	
NEG	-	153.0912	7.24	-	x		x	
POS	152.0848	135.0815	7.24	-	x		x	
POS	180.0799	181.0850	7.24	-	x	x	x	
POS	-	449.2116	7.24	-	x		x	
POS	-	191.1040	7.24	-	x		x	
POS	-	237.0443	7.24	-	x	x		
NEG	-	205.0499	7.26	-	x		x	
POS	-	330.2256	7.27	-	x	x	x	x
POS	-	293.1383	7.27	-	x	x	x	
NEG	173.1024	218.1018	7.30	-	x		x	
POS	-	109.1018	7.30	-	x		x	

NEG	-	287.1476	7.31	-	x		x	
POS	150.1049	151.1122	7.31	-	x		x	x
POS	-	259.1310	7.31	-	x		x	
NEG	-	169.1218	7.33	-	x		x	
POS	194.0925	217.0812	7.33	-	x		x	
POS	-	195.1041	7.33	-	x		x	
POS	-	237.1127	7.33	-	x		x	x
POS	-	232.0889	7.34	-	x		x	
POS	-	473.2284	7.34	-	x		x	
POS	-	242.1213	7.41	-	x	x		
NEG	-	175.0963	7.42	-	x		x	
NEG	-	279.1340	7.45	-	x		x	
NEG	-	273.1692	7.46	-	x		x	
POS	-	137.0973	7.48	-	x		x	
POS	-	149.0972	7.49	-	x		x	
POS	-	291.1208	7.49	-	x	x	x	x
POS	-	358.2569	7.51	-	x			x
POS	-	193.0872	7.51	-	x	x	x	
POS	-	422.2488	7.52	-	x		x	
NEG	-	429.1936	7.57	-	x			x
POS	-	389.2310	7.57	-	x		x	
NEG	-	185.1168	7.59	-	x		x	x
POS	226.1187	227.1260	7.59	-	x		x	
NEG	-	427.1777	7.60	-	x		x	
POS	-	109.1019	7.60	-	x			x
POS	-	476.2539	7.60	-	x		x	
POS	-	153.0902	7.62	-	x		x	
POS	-	221.1189	7.62	-	x		x	x
POS	-	460.1405	7.62	-	x		x	
POS	-	295.1780	7.62	-	x		x	
NEG	154.0982	153.0908	7.63	-	x		x	
NEG	-	219.0633	7.63	-	x		x	
POS	-	475.2462	7.63	-	x		x	
NEG	-	191.0329	7.66	-	x		x	
NEG	-	443.1734	7.69	-	x		x	x
POS	-	381.1602	7.69	-	x		x	
NEG	-	511.1591	7.70	-	x		x	x
POS	-	477.2557	7.70	-	x		x	
POS	-	289.1395	7.70	-	x			x
POS	-	175.1123	7.73	-	x		x	

POS	-	193.1250	7.74	-	x	x		x
NEG	-	137.0956	7.75	-	x		x	
POS	270.1434	293.1370	7.75	-	x			x
POS	-	294.1467	7.75	-	x		x	
NEG	158.1316	203.1280	7.78	-	x	x	x	
NEG	-	211.0958	7.78	-	x	x		
POS	-	257.1069	7.78	-	x		x	x
NEG	204.1350	185.1171	7.79	-	x		x	
POS	-	257.1152	7.79	-	x		x	
POS	-	141.1282	7.79	-	x		x	x
POS	-	475.2464	7.79	-	x		x	
POS	-	227.1265	7.79	-	x		x	
NEG	-	459.1677	7.81	-	x		x	x
POS	-	225.0874	7.81	-	x		x	
POS	-	189.1258	7.81	-	x			x
POS	-	215.1000	7.82	-	x		x	
POS	-	221.1174	7.82	-	x		x	
POS	-	307.1182	7.85	-	x		x	
POS	-	371.2143	7.85	-	x		x	
NEG	114.1038	113.0965	7.86	-	x		x	
POS	-	309.1669	7.86	-	x	x	x	
NEG	-	323.1155	7.88	-	x			x
POS	-	137.0978	7.88	-	x		x	
POS	-	309.1576	7.88	-	x		x	
POS	-	298.1479	7.88	-	x		x	
POS	-	271.0874	7.88	-	x		x	
NEG	-	155.1090	7.90	-	x		x	
NEG	-	497.1806	7.90	-	x			x
POS	-	201.0919	7.90	-	x		x	
POS	-	267.1195	7.90	-	x		x	
POS	-	306.1382	7.90	-	x		x	
POS	-	288.1133	7.92	-	x	x		
NEG	-	209.1176	7.95	-	x		x	
POS	-	133.1037	7.95	-	x		x	
NEG	-	243.1215	7.96	-	x		x	
POS	-	147.0831	7.96	-	x		x	
POS	-	387.2076	7.96	-	x		x	x
POS	-	133.1020	7.96	-	x		x	x
NEG	-	181.0853	7.97	-	x			x
POS	-	393.2207	7.97	-	x		x	

POS	234.0913	257.0761	7.99	-	x		x	
POS	254.0584	255.0657	7.99	-	x		x	
POS	-	239.0597	7.99	-	x	x	x	
POS	-	109.1018	7.99	-	x		x	
POS	-	195.0705	7.99	-	x		x	
POS	-	339.0174	7.99	-	x		x	
POS	-	371.1369	8.00	-	x	x		
POS	-	137.0958	8.00	-	x		x	
POS	-	188.0373	8.00	-	x		x	
NEG	-	223.1341	8.02	-	x		x	
POS	252.1335	275.1247	8.02	-	x	x	x	x
NEG	-	251.1267	8.06	-	x		x	
POS	240.1347	263.1212	8.06	-	x	x		
POS	-	274.0952	8.08	-	x		x	
NEG	-	299.0563	8.10	-	x		x	
POS	-	327.1635	8.10	-	x		x	x
POS	-	237.1496	8.11	-	x		x	
POS	-	327.1724	8.12	-	x		x	
NEG	-	648.1454	8.14	-	x		x	
POS	298.1257	321.1109	8.14	-	x		x	
POS	-	355.0523	8.14	-	x		x	
POS	-	121.1013	8.14	-	x		x	x
POS	-	107.0500	8.15	-	x		x	
POS	-	257.1229	8.19	-	x		x	
POS	-	243.1010	8.19	-	x		x	
POS	-	149.0614	8.21	-	x		x	
POS	-	123.0448	8.21	-	x		x	
POS	-	133.0653	8.21	-	x		x	
POS	-	343.1499	8.24	-	x		x	
NEG	-	139.1120	8.26	-	x		x	
POS	-	247.0959	8.28	-	x	x		x
POS	-	139.1118	8.28	-	x		x	x
POS	-	373.1937	8.29	-	x		x	
NEG	-	229.0167	8.32	-	x		x	
POS	-	333.1186	8.32	-	x			x
POS	-	287.1271	8.33	-	x			x
POS	-	135.1173	8.35	-	x		x	
POS	-	270.1463	8.35	-	x	x		
POS	-	123.0813	8.36	-	x		x	
POS	-	107.0861	8.36	-	x	x		

POS	-	323.1792	8.36	-	x	x		
NEG	-	247.0950	8.37	-	x		x	
NEG	-	169.1223	8.37	-	x		x	x
POS	-	145.1021	8.37	-	x		x	
POS	-	153.1278	8.37	-	x	x		
POS	-	163.1119	8.39	-	x		x	
NEG	-	225.1121	8.40	-	x		x	
NEG	-	151.1119	8.40	-	x		x	
NEG	-	163.1112	8.40	-	x		x	
NEG	-	293.1017	8.40	-	x		x	
NEG	-	213.1129	8.40	-	x		x	
POS	-	379.2408	8.40	-	x		x	
POS	-	237.1156	8.40	-	x		x	
POS	-	237.1118	8.40	-	x		x	
POS	-	167.1038	8.40	-	x		x	
POS	-	395.2411	8.40	-	x		x	
POS	-	137.0950	8.40	-	x			x
POS	166.0992	149.0959	8.41	-	x		x	x
POS	-	149.0927	8.41	-	x		x	
POS	-	137.0967	8.41	-	x		x	
NEG	-	361.2011	8.44	-	x		x	x
POS	-	323.1748	8.44	-	x		x	
POS	-	325.1974	8.44	-	x			x
NEG	-	363.1808	8.45	-	x		x	x
POS	384.1915	385.1988	8.45	-	x		x	x
POS	-	363.2166	8.45	-	x		x	x
POS	-	345.2021	8.45	-	x		x	
POS	-	303.1503	8.48	-	x		x	
POS	-	401.2824	8.48	-	x		x	
NEG	252.1348	251.1275	8.50	-	x		x	
NEG	272.1359	293.1397	8.51	-	x		x	x
NEG	-	307.1210	8.51	-	x		x	
POS	-	387.2185	8.51	-	x		x	
NEG	-	361.1281	8.52	-	x		x	x
NEG	-	225.0732	8.52	-	x			x
POS	276.1319	277.1421	8.52	-	x	x	x	x
POS	-	333.1645	8.52	-	x		x	
POS	-	317.1386	8.54	-	x		x	
NEG	224.1405	223.1332	8.58	-	x		x	x
POS	-	341.1502	8.59	-	x			x

NEG	-	339.1258	8.62	-	x	x	x	x
POS	-	293.1380	8.62	-	x		x	
NEG	-	225.1486	8.63	-	x		x	
NEG	-	389.0924	8.63	-	x		x	
NEG	238.1555	237.1482	8.66	-	x		x	
NEG	292.1872	273.1693	8.68	-	x		x	
NEG	348.1911	347.1833	8.68	-	x		x	
NEG	258.1801	257.1728	8.68	-	x		x	
NEG	-	315.1956	8.68	-	x		x	
NEG	-	383.1829	8.68	-	x		x	
POS	-	297.1730	8.68	-	x		x	x
POS	-	383.1893	8.68	-	x		x	
POS	-	361.1999	8.68	-	x		x	x
POS	-	445.1599	8.68	-	x			x
POS	-	281.1725	8.68	-	x		x	
POS	-	337.1692	8.68	-	x			x
NEG	-	427.1771	8.69	-	x		x	
POS	-	293.1745	8.69	-	x	x		
NEG	-	405.1875	8.70	-	x		x	x
POS	-	451.1777	8.70	-	x		x	x
NEG	476.2214	497.1809	8.72	-	x		x	
NEG	-	331.1145	8.72	-	x		x	
NEG	-	247.0942	8.73	-	x		x	
NEG	-	359.1151	8.74	-	x		x	x
POS	-	347.1290	8.76	-	x		x	x
POS	-	263.1602	8.76	-	x			x
NEG	216.1344	237.1103	8.77	-	x		x	x
NEG	-	153.1268	8.77	-	x		x	
NEG	-	379.2081	8.77	-	x		x	
NEG	-	340.1545	8.77	-	x	x	x	
NEG	-	231.1020	8.80	-	x			x
POS	-	295.1539	8.80	-	x	x	x	
POS	-	277.1381	8.81	-	x		x	
POS	-	257.1575	8.81	-	x		x	x
NEG	-	267.1585	8.83	-	x		x	
POS	276.1339	299.1257	8.83	-	x		x	
POS	-	291.1558	8.83	-	x		x	
POS	-	231.1355	8.83	-	x		x	
POS	-	317.1367	8.83	-	x	x	x	
POS	-	279.1529	8.84	-	x		x	

POS	-	169.1044	8.84	-	x		x	x
NEG	-	361.1981	8.85	-	x		x	
NEG	-	291.1258	8.87	-	x		x	
POS	-	345.1952	8.87	-	x		x	x
NEG	-	269.1374	8.88	-	x	x	x	x
NEG	-	399.1451	8.90	-	x		x	
POS	-	179.1440	8.90	-	x			x
NEG	248.1068	293.1078	8.94	-	x			x
NEG	-	198.1127	8.94	-	x		x	x
POS	-	270.1126	8.94	-	x		x	
POS	-	292.0984	8.95	-	x		x	
POS	-	401.2900	8.98	-	x		x	x
NEG	-	183.1372	9.01	-	x		x	
NEG	228.1354	249.1100	9.03	-	x		x	x
POS	322.2187	345.2020	9.10	-	x	x	x	
POS	-	345.1982	9.10	-	x		x	
POS	-	349.2293	9.18	-	x		x	
NEG	-	459.2046	9.21	-	x		x	
POS	-	354.1380	9.23	-	x		x	
NEG	-	293.1413	9.24	-	x	x	x	x
POS	-	235.1321	9.27	-	x		x	
POS	-	383.2804	9.27	-	x		x	x
NEG	-	363.2168	9.29	-	x		x	x
NEG	-	431.2039	9.29	-	x		x	x
POS	346.2148	347.2217	9.29	-	x		x	x
POS	-	269.1803	9.29	-	x			x
POS	-	352.1209	9.31	-	x		x	
POS	-	293.1817	9.31	-	x		x	x
POS	-	387.2141	9.31	-	x		x	x
NEG	-	379.1760	9.34	-	x		x	
NEG	-	345.2122	9.34	-	x		x	
NEG	423.1710	422.1637	9.35	-	x		x	
POS	-	446.1653	9.35	-	x		x	
NEG	-	341.1059	9.36	-	x	x	x	x
NEG	-	229.0169	9.36	-	x		x	
NEG	-	413.1960	9.42	-	x			x
NEG	-	343.0851	9.42	-	x		x	
NEG	450.1981	495.1895	9.46	-	x		x	
NEG	-	501.2091	9.46	-	x		x	
NEG	-	433.2199	9.46	-	x		x	

POS	-	405.1960	9.46	-	x		x	
POS	-	275.1619	9.61	-	x		x	
NEG	-	354.1359	9.72	-	x		x	
POS	-	355.1491	9.73	-	x		x	x
NEG	-	455.1701	9.91	-	x		x	
NEG	-	387.1820	9.94	-	x		x	
POS	-	359.1845	9.98	-	x		x	
POS	-	349.2350	10.00	-	x		x	
NEG	-	385.1681	10.08	-	x		x	
NEG	-	265.1438	10.12	-	x		x	x
NEG	-	333.1701	10.16	-	x		x	x
POS	346.2495	347.2220	10.16	-	x		x	
POS	-	298.1335	10.46	-	x		x	
POS	440.3638	423.3605	10.55	-	x		x	
NEG	-	313.2379	10.74	DiHOME	x			
NEG	-	293.2109	11.05	-	x		x	
NEG	296.2345	295.2271	11.32	EpOME	x		x	
NEG	-	363.2146	11.32	-	x		x	
POS	-	339.1931	11.36	-	x			x
POS	-	317.2080	11.36	roxyretinoic	x			x
POS	-	341.2092	11.48	-	x	x	x	x
POS	296.2341	319.2236	11.66	EpETE	x		x	x
POS	-	321.1830	12.02	-	x		x	x
POS	-	297.1812	12.06	-	x		x	
NEG	-	301.2165	12.24	-	x		x	x
POS	-	357.1531	12.24	-	x			x
POS	-	341.1788	12.24	-	x		x	
POS	-	347.1954	12.24	-	x		x	x
POS	-	623.4025	12.25	-	x		x	x
NEG	-	277.2163	12.27	-Linolenic ac	x		x	x
NEG	-	345.2040	12.27	-	x		x	x
NEG	-	475.1619	12.27	-	x		x	
NEG	-	407.1743	12.27	-	x		x	
POS	368.1642	333.1504	12.27	-	x		x	
POS	278.2248	279.2321	12.27	-Linolenic ac	x		x	x
POS	-	691.3913	12.27	-	x			x
POS	-	225.0430	12.27	-	x		x	x
POS	-	617.3866	12.27	-	x		x	x
POS	-	137.1334	12.27	-	x			x
POS	-	123.1168	12.27	-	x		x	x

POS	-	317.1790	12.27	-	x	x	
POS	-	323.1963	12.27	-	x	x	
POS	350.2206	351.2279	12.46	-	x	x	x
POS	-	435.1817	12.46	-	x	x	x
POS	-	329.2514	12.46	-	x	x	x
POS	280.2415	263.2370	12.51	-	x	x	x
POS	150.1054	133.1021	12.51	-	x	x	x
POS	178.1370	161.1337	12.51	-	x		x
POS	-	135.1169	12.51	-	x	x	x
POS	-	599.4339	12.51	-	x	x	
POS	-	387.1818	12.51	-	x	x	
POS	-	627.4327	12.51	-	x	x	
POS	-	325.2116	12.51	-	x	x	
POS	-	179.1802	12.51	-	x	x	x
NEG	-	279.2319	12.53	-	x	x	x
NEG	-	379.1574	12.53	-	x	x	x
NEG	-	581.4549	12.53	-	x	x	x
NEG	-	409.1902	12.53	-	x	x	
NEG	-	649.4426	12.53	-	x	x	x
NEG	-	717.4297	12.53	-	x	x	
NEG	-	551.1824	12.53	-	x	x	
POS	180.1516	163.1483	12.53	-	x	x	x
POS	164.1202	147.1169	12.53	-	x	x	x
POS	166.1354	149.1321	12.53	-	x	x	x
POS	672.3979	695.4205	12.53	-	x	x	
POS	638.4222	621.4189	12.53	-	x	x	
POS	370.1804	335.1666	12.53	-	x	x	
POS	138.1029	121.0997	12.53	-	x	x	x
POS	136.0894	119.0861	12.53	-	x	x	x
POS	192.1530	175.1497	12.53	-	x	x	x
POS	206.1680	189.1647	12.53	-	x	x	x
POS	560.4787	561.4903	12.53	-	x	x	
POS	-	339.1645	12.53	-	x		x
POS	-	137.1325	12.53	-	x	x	x
POS	-	123.1169	12.53	-	x	x	x
POS	-	336.1720	12.53	-	x	x	
POS	-	337.1692	12.53	-	x	x	
POS	-	461.1855	12.53	-	x	x	
POS	-	109.1018	12.53	-	x	x	
POS	-	320.1989	12.53	-	x	x	

POS	-	151.1490	12.53	-	x		x	x
POS	-	221.2260	12.53	-	x		x	x
POS	-	170.9962	12.53	-	x		x	
POS	-	81.0704	12.53	-	x		x	x
POS	-	227.0389	12.53	-	x		x	
POS	-	239.0584	12.53	-	x		x	
POS	550.4252	551.4325	12.75	-	x		x	
POS	-	271.2631	12.76	-	x		x	x
NEG	-	281.2479	12.88	-	x		x	x
NEG	-	349.2354	12.88	-	x		x	x
NEG	-	411.2050	12.88	-	x		x	x
POS	264.2459	265.2520	12.88	-	x		x	
POS	344.2297	327.2264	12.88	-	x		x	
POS	-	631.4667	12.88	-	x		x	
POS	-	395.2173	12.88	-	x		x	
POS	-	339.1848	12.88	-	x		x	
POS	-	389.1996	12.88	-	x		x	
POS	-	337.1821	12.88	-	x		x	
POS	-	321.2116	12.88	-	x		x	
POS	-	353.2368	12.88	-	x		x	x
POS	358.3089	381.2979	12.91	-	x		x	
POS	-	463.2017	12.91	-	x		x	
POS	-	378.7907	12.93	-	x		x	
POS	-	131.0031	12.93	-	x		x	
POS	-	299.1427	13.70	-	x	x		
POS	-	607.2530	13.70	-	x	x		
