# Supplementary figures and images for: Mechanism of Chronic Kidney Disease Progression and Novel Biomarkers: A Metabolomic Analysis of Experimental Glomerulonephritis
Source: Metabolites. 2020 Apr 24;10(4):169. doi: 10.3390/metabo10040169 (PMC7240957; doi:10.3390/metabo10040169)

Figure S1.

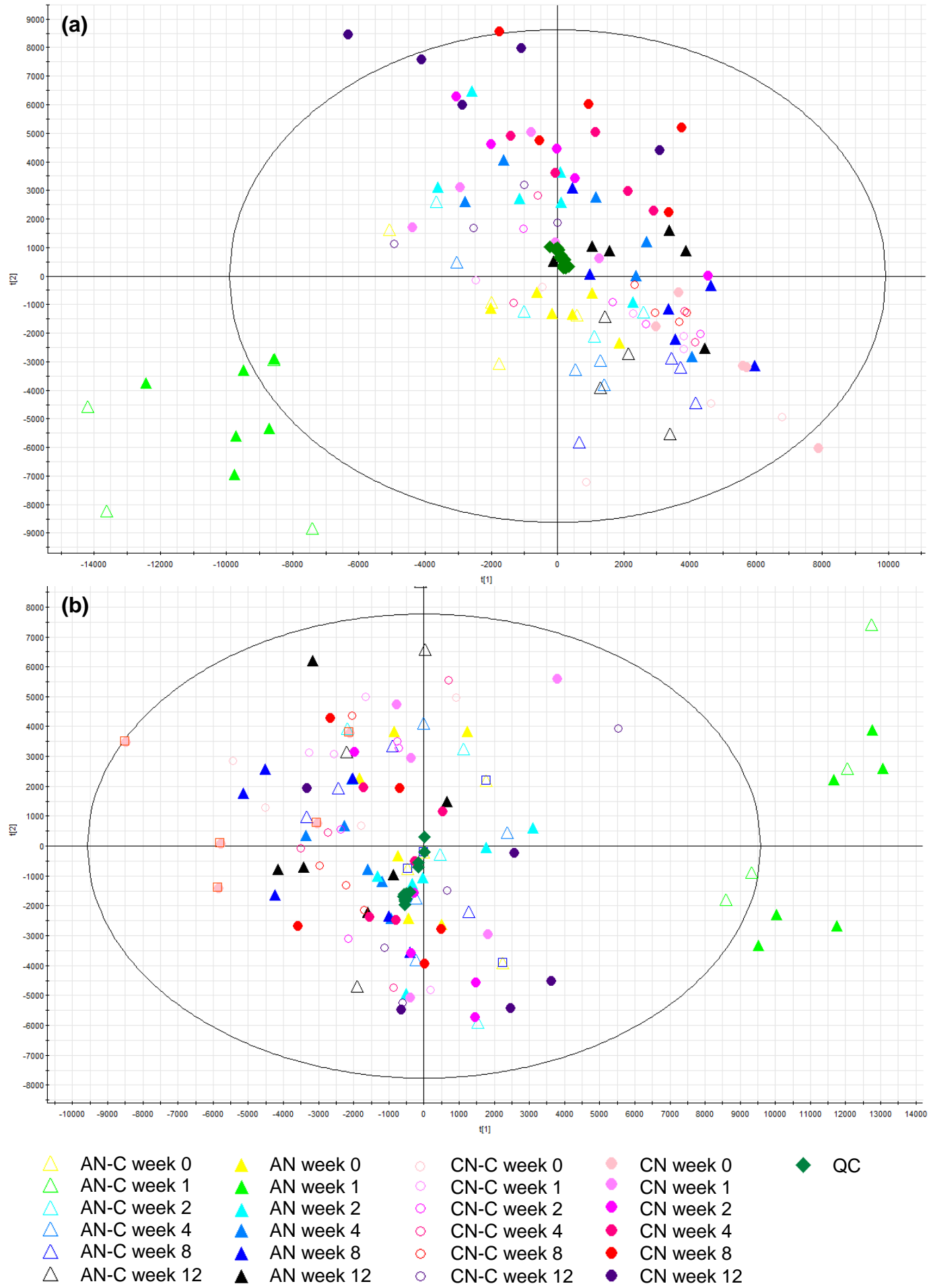

Supplement: Supplementary file 1 [file metabolites-10-00169-s001.zip › Supplementary Figure S1.pdf]
